# The rise in trauma & orthopaedic trainee-led research and audit collaborative projects in the United Kingdom since the start of the COVID-19 pandemic

**DOI:** 10.12669/pjms.39.3.7417

**Published:** 2023

**Authors:** Tahir Khaleeq, Rakan Kabariti, Usman Ahmed

**Affiliations:** 1 Tahir Khaleeq, MBBS, MRCSEd, Pg Dip MedEd Trauma & Orthopaedic Department, Princess Royal Hospital, Shrewsbury and Telford NHS Trust, Telford, United Kingdom; 2 Rakan Kabariti, PGcert (Leadership), PGDip (Meded), MBChB, MRCS Trauma & Orthopaedic Department, Princess Royal Hospital, Shrewsbury and Telford NHS Trust, Telford, United Kingdom; 3 Usman Ahmed, MBBS, Phd., FRCS, FRCS Trauma & Orthopaedic Department, Princess Royal Hospital, Shrewsbury and Telford NHS Trust, Telford, United Kingdom

**Keywords:** Trauma and Orthopaedics, Trainee-led Collaborative Projects, Research and Audit Collaborative projects

## Abstract

**Background and Objective:**

A significant increase has been observed globally in multi-centre trainee-led trauma & orthopaedic (T&O) research collaborative projects with more emphasis have been on tackling important research questions since the start of the COCID-19 pandemic. The objective of our analysis was to determine the number of trainee-led research collaborative projects in T&O in the United Kingdom that were started during the COVID-19 pandemic.

**Methods:**

A retrospective analysis was conducted to determine how many trainee-led national collaborative projects in T&O were conducted since the start of the COVID-19 pandemic lockdown (March 2020 to June 2021) and the number of projects identified were compared to the previous year (2019). Any regional collaborative projects, projects that were started before the onset of COVID and projects of other surgical specialities were not included in the study.

**Results:**

There were no projects identified in 2019 while in the Covid pandemic lockdown we identified 10 trainee-led collaborative trauma & orthopaedic projects with six of them being published with level of evidence from three to four.

**Conclusion:**

Covid was unprecedented and has placed considerable trials across healthcare. Our study highlights an increase in multi-centre trainee-led collaborative projects within the UK and it underlines the feasibility of such projects especially with the advent of social media and Redcap® which facilitate recruitment of new studies and data.

## INTRODUCTION

During the previous year, there has been an upsurge of trainee-led Orthopaedic research collaborative projects globally. The concept of Trainee Led Collaborative projects is not a new concept in the UK and one such project was a two-year study in the 1980s looking at measles which was conducted by the Royal College of Surgeons of General Practitioners trainees.^1^ Surgical trainee collaboratives are trainee bodies, which are run by medical students/ training junior doctors, who collaborate across the country to embark on multicentre research. Such projects are a good opportunity to bring together trainees/ medical students across the country to work together in answering clinically important questions.

The recent pandemic has been a driving force for an increase in the number of collaborative projects in the United Kingdom. There has also been an increase in the enthusiasm of the trainees to get involved in research and to collectively answer important clinical questions. The National Research Collaborative (NRC) was established which acts as an umbrella organisation which facilitates multiple collaborative groups and networks and aims to promote participation among various specialities, including general surgery and Trauma and Orthopaedics. To promote this, the NRC has guides that aim to help set up projects that are collaborative focusing on the key operational and organizational principles.^2^ Among all of this lies a committee with active trainee participation, effective communication and endorsement of national medical bodies.

The value and importance of the recent rise of successful Orthopaedic trainee Led research projects in the past year are always discussed with arguments both for and against.^3^,^4^ To date, there has been no factual study which analyses the research activity, productivity and true impact of Trainee-led Trauma and Orthopaedic Collaborative researches which aim to inform healthcare services in other countries

In this study, we aim to evaluate the number of trauma & orthopaedic trainee-led research collaborative projects that took part since the start of the COVID-19 pandemic in the UK, exploring the value and feasibility of such collaboratives in driving forwards clinical academia.

## METHODS

A systematic search was done online using key phrases ‘trainee research’, ‘trainee-led collaborative’ and ‘Orthopaedic trainee-led research collaborative’. Websites such as Association of Surgeons in training (ASIT), Twitter, British Orthopaedic Training Association (BOTA) and British Orthopaedic Association (BOA) were also checked for any trainee-led collaborative projects that were posted.^5^,^6^ All the Collaborative Projects included in this study had their websites reviewed and committee conducting the research contacted for further data. To assess the evidence emerging from trainee-led collaborative research the ‘levels of evidence’ by Oxford Centre for Evidence Based Medicine was used.^7^ In this measure the evidence is from five (which is the lowest and is an expert opinion) to 1a (which is the highest and includes research such as systematic review of Randomized Control Trials).

### Duration:

The timeline for data collection had been from March 2020 to January 2021, essentially the Covid Lockdown period in the United Kingdom.

### Place of Study:

All of the projects identified in this study were done in the United Kingdom.

### Exclusion Criteria:

- Any regional collaborative projects, projects that were started before the COVID-19 and projects that involved other surgical specialities were excluded from the study.
- The number of projects identified was also compared to that published in 2019.
- All conference abstracts and proceedings were also excluded from the analysis.

### Inclusion Criteria:

- All of the publications were identified from website listings of collaboratives and searches done on PubMed using the project names.
- All of the publications which were PubMed-indexed were included in the study.

### Ethical Approval:

Using the Health Research Authority decision tool, this study was not deemed to need ethical approval.

## RESULTS

Ten trainee-led collaborative trauma & orthopaedic projects were included in the analysis with six being published in peer reviewed journals. The level of evidence ranged between three and four. There were five audits and five cohort studies. The patients that were included in the studies ranged from 927 to 140,231 with Supraman Collaborative study with the lowest number of recruited patients.

Comparing to 2019, we were not able to identify any trainee-led collaborative projects. Collectively 2249 centres participated in collaboratives projects with the maximum number of centres participating in one study was 1674 and lowest number was 38 centres. Almost all of the collaboratives recruited centres by the use of social media, twitter, or by networking in conferences and the use of the British Orthopaedic Trainee Association.

**Fig.1:**
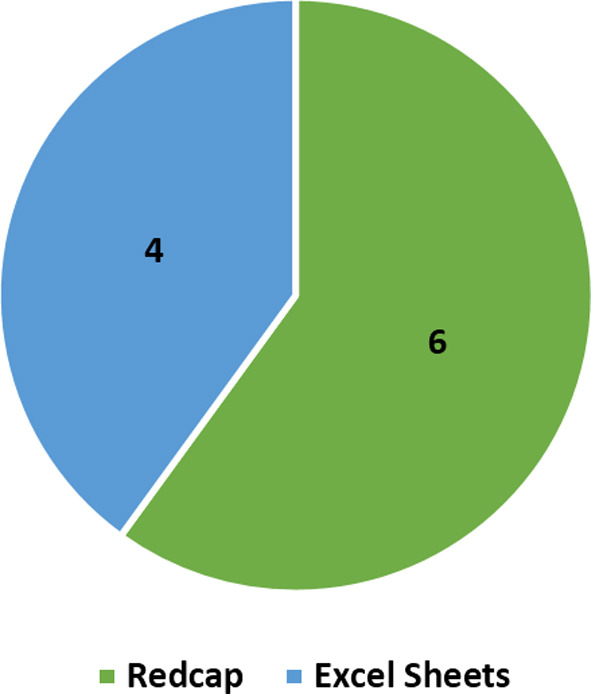
Data Collection Technique.

The data collection method used most was by using REDCAP (Research Electronic Data Capture), which is a web application which manages databases and online surveys in a secure encrypted manner, and confidential excel sheets were also used (Fig.1).

### COVID Surg:

COVID Surg Collaborative Surg Week was a cohort study which was international, multicentre, and prospective in nature and included patients undergoing any type of surgery. This was initiated by the NIHR Global health research unit on Global surgery, Birmingham UK and the aim of the study was to get more data as to update clinical practice during the Covid Pandemic regarding the importance of identification of symptomatic vs. asymptomatic SARS-COV-2 preoperatively along with the determination of the optimal timing of surgery following an infection. One Hundred forty thousand two hundred and thirty one patients were included in the study with 1674 hospitals in 116 countries. Data was collected up to four blocks of seven consecutive days (5 October 2020 to November 1^st^ 2020) and collected using a secure online database (Redcap). Conclusion of the study was as there was a higher mortality in patients after seven week delay with ongoing symptoms compared to those patients who were asymptomatic and whose symptoms had resolved. This research resulted in four PubMed cited publications in various high impact journals.^8^-^11^

### FFPOM:

Fragility fracture post operative mobilization was a national Collaborative with an aim to establish the understanding of current practice of post operative weight bearing for lower extremity fragility fractures. It was comparing to the British Orthopaedic Association Standards’ The care of the older of frail orthopaedic trauma patient was published in May 2019. The standard mentioned that all frail patients after their surgical procedure should be allowed to weight bear fully within 36 hours of admission.^12^ Inclusion Criteria was any patient who was more than 60 year old with a lower limb fracture that was managed operatively. Exclusion criteria were non-operatively managed fractures, high energy injured, polytrauma and pathological fractures.

Data was collected prospectively and retrospectively from 1st January to 30th June 2019 (retrospective) and 1st February to 14th March 2021 (prospective). Collection of data was done on a Excel spreadsheet and analysis of the data was done against the BOA standard (Full weight bearing for activities of daily living). Hospital-by-hospital variation was calculated for fracture location and description, type of operation, seniority of operating surgeon and duration of restricted weight bearing. Nineteen thousand one hundred fifty three patients were included in the study with 81 hospitals taking place with 430 collaborators. The Conclusion of the study was that there was a large difference noted in the overall percentage of Neck of femur fractures vs non neck of femur fractures fragility fracture patients that were allowed to fully weight bear after surgery. Outside of the femur, the most common fracture locations for non-Neck of femur fragility fractures are foot and ankle fractures and proximal tibia fractures. This audit was published with a proposal for further research to enable surgeons to feel confident to make non-Neck of femur fragility fractures to become Fully weight bear after surgery.

### CIPHUR:

Chlorhexidine Gluconate versus Povidone-Iodine Skin Antisepsis Prior to Upper limb Surgery (CIPHUR) was a prospective National service evaluation. The aim was to compare local practise of antiseptic use and compare them to the standards outlined by NICE which recommended using alcoholic Chlorhexidine gluconate (CHG) for preoperative skin preparation to reduce the risk of Surgical site infections. The inclusion criteria were any adult or children identified prior to any form of surgery distal to the shoulder joint while the exclusion criteria were any active infection at the time of upper limb surgery. The audit included 2,454 patients and collaborators from 92 centres participated and data was recruited via Excel sheets. The Systematic review and network meta- analysis of antiseptic in clean surgery and the national survey of clinical practice and clinician opinion was done and is pending publication.

### Emprove:

Evaluating the Measures in patient reported outcomes, values and experiences (emprove) was a national collaborative audit of elective orthopaedic clinical practise conducted by the south west orthopaedic division (SWORD). The audit was against national society standards with the aim to assess concordance with standards and define the configuration of current PROMS ***(**Patient related outcome measures**)*** practice within elective orthopaedics. 38 enrolled trusts across nine regions participated in the national audit. The conclusion of the audit was that standardization of PROMS practice is required across all orthopaedic procedures as there is limited consensus and wide variation in their usage. However, the integration of PROMS within Best Practise Tariff has encouraged PROMs uptake and consistency. The audit has not been published yet.

### Supraman:

Supracondylar fracture management was a retrospective trainee led national evaluation which was designed and led by members of the South West Orthopaedic Research division (SWORD). The primary objective was to identify the surgical practice and post-operative care being provided for displaced supracondylar elbow fractures across the United Kingdom. Secondary objectives included the identification of patient characteristics, common mechanisms of injury, fracture types undergoing surgical intervention, and significant post-operative complications associated with the management of these injuries. Recruitment was done across the United Kingdom through promotion of Twitter. Inclusion criteria were patients that were less than 16 years of age with a displaced supracondylar fracture of the elbow of any time confirmed on X-ray that required acute surgical intervention between the 1^st^ of January 2019 of December 2019. Exclusion criteria were open fractures, undisplaced fractures, if there was a separate acute but distinct fracture present on the ipsilateral upper limb or if primary surgical intervention occurred over three weeks from the time of injury. Data collection was done via Redcap. Nine hundred twenty seven patients were identified as undergoing surgical intervention for a displaced supracondylar elbow fracture across 42 hospitals. The conclusion of the study was that the majority of displaced supracondylar fractures of the elbow are operated on during daytime hours, with most being performed the day after injury. Varying surgical techniques are utilised across the United Kingdom. Overall, two wires in a cross configuration were most commonly utilised with the wires left percutaneous. However, Paediatric Orthopaedic specialists appear to prefer lateral only fixation. A very low rate of deep infection and revision surgery for displacement was noted in both crossed and lateral only fixation patients. A manuscript has been prepared and sent to a journal for consideration of publication.

### Suspected Scaphoid:

It was a cross sectional study which was survey based conducted in 87 centres in the UK from November 2020 to April 2021. The aim of the study was to investigate the availability of radiological imaging (MRI) directly from the Emergency Department and minor injury units and whether pathways were made for suspected scaphoid fractures. Recruitment was done via previous projects, social media (twitter) and clinician contacts. All centres that regularly treated acute wrist trauma were included in the study. The study showed that only a fraction of hospitals across the UK offers MRI directly from minor injury units and emergency departments when suspecting a scaphoid fracture. The results of the study have been published in the Bone and joint journal on the Nov. 29, 2021.^12^,^13^

### HARNT Impact:

Hindfoot ankle reconstruction Nail trail (HARNT) was a national collaborative study in affiliation with British Orthopaedic Trainee Association and data was collected retrospectively between 1^st^ January to June 30^th^ 2019. The main objective was to determine the outcomes and management of complex ankle fractures in the United Kingdom and compare them to the BOAST guidelines for the management of complex ankle fractures. All adult patients with complex ankle fractures (Ao43/44) which were open or closed were included in the study. These complex fractures included patient with diabetes with or without neuropathy, rheumatoid arthritis, alcoholism, polytrauma and cognitive impairment. Fifty-six centres participated in the study and data from 1360 patients were collected. The study concluded that 9% of patients were managed with a Hind foot nail and were also noted to have the most complications. Only 21% of patients were allowed to weight bear fully after the procedure despite BOAST guidance. The Results have not been published.

### BASK EPPIC

Evaluation of practice patellofenoral instability collaborative was a BASK trainee collaborative retrospective national audit that was performed over a five years period. The Audit was to evaluate which procedures and in which combination are being used to surgically manage patellofemoral instability in the United Kingdom and these were compared to the British Orthopaedic Association surgical management of recurrent patellar instability.

Data was collected by the help of coding departments and theatre records and were analysed and on excel sheets. Fifty sites across the United Kingdom participated with 3,639. The study showed that the surgical management of Patellofemoral instability varies across the country but as new national guidelines are implemented a re-audit of practice should be done. This has not been published.

### PanSurg PREDICT:

Pansurg Predict was an International Observational Cohort study which was retrospective and prospective in nature. This study was sponsored primarily by Imperial College London with the primary aim to measure the risk associated with patients presenting to hospital with a surgical pathology during the pandemic. Secondary aim was to create a dynamic risk prediction model. Collaborators were recruited via social media, Twitter and Data collection was done by REDCAP.

**Table-I:** Results.

Study	Retrospective/ prospective	Population	Participating centres	Published
COVID Surg	Prospective	140231	1674	Y
FFPOM	Prospective and Retrospective	19153	81	Y
CIPHUR	Prospective	2454	92	N
Emprove	Retrospective		38	N
Supraman	Retrospective	927	42	N
Suspected Scaphoid	Prospective	0	87	Y
HARNT Impact	Retrospective	1360	56	N
EPPIC	Retrospective	3639	50	N
PanSurg PREDICT	Prospective and Retrospective	3176	55	Y
Corona Hands	Prospective	1093	74	Y

Data was collected retrospectively and prospectively and the data was collected in such a way that it coincided with the publications by the Royal Colleges of Surgeons Guidance for surgeons working during COVID-19 pandemic on 20^th^ March 2020, 5^th^ April 2020, 20^th^ May 2020 and 26^th^ June 2020.

All patients who presented to hospitals with an acute orthopaedic pathology during 9^th^ March 2020 to30th August 2020 were included in the study. There were 55 Participating centres from 18 countries.

The study showed that the capacity of the operating room declined by 63.6% along with a decline of surgical staff by 27.2%. The results were published in the Annals of Surgery on 2021.^14^

### Corona Hands:

This was a national, multicentre observational cohort study with an aim to assess the safety of upper extremity surgery and to assess the 30 days mortality of patients. The secondary objectives were any complications related and unrelated to SARS COV-2 and any hospital safety processes that were in place. Data collection was between 01/04/2020 to 14/04/2020. The collaborators were recruited by social media and data was collected on a standardised encrypted excel spreadsheet. About 74 centres participated in the study with 1093 patients being recruited into the study. The study showed that Complications related to Sars-cov-2 were 0.18% for upper limb surgery with zero deaths when patients were operated on the same day as admission.

The study also suggested that surgery should not be delayed whilst waiting for the results of the Sars-Cov-2 test for any upper limb day case surgery. The study was published in the BMJ quality and safety Journal on April 2021.^15^

## DISCUSSION

There is little literature available highlighting the importance of Trainee led collaborative research projects and no scientific analysis such as our study has been carried out in the past. Our analysis provides the current direction and activity within the UK trainee collaborative movement however even though larger studies with multiple centres and high recruitment of patients are being undertaken we have noted that only six studies have contributed to the literature by publishing in peer review journals.

Our Analysis is suggestive of an increased ambition of the various groups in the UK with more prospective/retrospective national studies. Multiple regional orthopaedic trainee organizations such as British Orthopaedic Training Association (BOTA), south west orthopaedic division (SWORD) have taken an initiative to ensure more multi-collaborative projects are started. These Trainee bodies are pivotal in promoting such collaborations in the United Kingdom and encourages the expansion of such collaboration in other specialties. Our study also highlights an expanding footprint in literature by these UK trainee collaborative research projects which are of respectable quality directly impacting clinical practise.^16^

The main contributory factors of the increase in UK trainee led collaborative projects are a highly ambitious trainee body within a postgraduate surgical training system boosted by the COVID Pandemic. We feel that the surge of such collaborative projects especially during the pandemic are due to the fact that the junior doctors understood the importance of being involved in high yield research and contributing to and improving the current evidence-based practise of common pathologies. We feel Covid was a trigger for junior doctors to get involved in more research and maybe this was due to the fact that there was less operating due to the COVID pandemic. An increased enthusiasm has also been noticed after the Royal College of Surgeons Clinical Trials Initiative have established a wide network of trial centrals across the United Kingdom.^17^

Our analysis also notes the importance of social media for recruitment and programs such as Twitter. These have been instrumental for the success of such multicentre collaborative projects as due to the pandemic there was less chances of networking in conferences, courses, workshops, and other events.

REDCAP is growing in popularity in the multicentre collaborative projects as it is a centralized online database. They provide an accessible, secure and affordable approach to database and statistician access and this ensures the long-term success of the trainee collaborative movement.

However, the most core aspect of the future success of more Multicentre collaborative projects of trainee led collaborative projects is ensuring the enthusiasm of the Trainees is maintained through open participation and ensuring fair recognition of the involvement of trainees. In all of projects included in the study the method of recognition has been the use of a collaborator status in peer reviewed journals.

The introduction of Collaborative Trainee led Research projects is essential across the world for trainees as they can develop and sharpen their research methodologies and especially in countries, like Pakistan, where trainees are not satisfied with their development of research skills.^18^,^19^ This will encourage the development of a research culture in Pakistan which in turn will encourage the improvement of training and attitudes towards research which will ensure to improve their understanding of evidence based medicine and also contribute to it.^20^

### Limitations:

The limitations of this study stem from the small sample size and the lack of representation from other countries. However, we feel that after this research is published and the advantages seen, there will be an increase of impact caused by Trainee led collaborative projects in Europe and worldwide.

## CONCLUSIONS

Orthopaedic Surgical trainees in the United Kingdom have been instrumental in the development of an innovative and valuable model for healthcare research despite COVID 19 having placed significant challenges. This is proven by the fact that there has been a significant increase in such collaborative Orthopaedic Trainee led projects in the United Kingdom in the Covid Lockdown.

### Authors’ contribution:

**TK:** Designed, Collected, analysed data, write up of paper.

**RK:** Analysis and review of paper

**UA:** Supervisor, final check and responsible and accountable for the accuracy or integrity of the work.
